# Subcutaneous Fat Is Associated with Improved Survival in Patients with Non-Metastatic Clear Cell Renal Cell Carcinoma: Dissecting the Obesity Paradox

**DOI:** 10.3390/cancers18162626

**Published:** 2026-08-14

**Authors:** Reza Lahiji, Susan Mumford, Benjamin N. Schmeusser, Gloria Fung, Charan Koltur, Baris Esen, Lorenzo Storino Ramacciotti, William Luke, Pooja Hemige, Valentina Grajales, Vikram N. Narayan, Reza Nabavizadeh, Mohammad Hajiha, Shreyas S. Joshi, Nazih Khater, Sarah P. Psutka, Kenneth Ogan, Viraj A. Master

**Affiliations:** 1 Department of Urology, Emory University School of Medicine, 1365 Clifton Road NE, Building B, Suite 1400, Atlanta, GA 30322, USA; reza.lahiji@emory.edu (R.L.); susan.mumford@downstate.edu (S.M.); gloria.fung@emory.edu (G.F.); nazih.khater@emory.edu (N.K.); kogan@emory.edu (K.O.); 2 Department of Urology, Indiana University School of Medicine, Indianapolis, IN 46202, USA; 3 Department of Urology, Koc University School of Medicine, Istanbul 34450, Turkey; 4 Department of Urology, Duke Cancer Center, Durham, NC 27710, USA; 5 Department of Urology, University of Washington, Seattle, WA 98195, USA; 6 Winship Cancer Institute, Emory University School of Medicine, Atlanta, GA 30322, USA

**Keywords:** visceral fat, subcutaneous fat, obesity paradox, CT segmentation, renal cell carcinoma

## Abstract

**Introduction:** The “obesity paradox” describes the observed association between improved cancer-specific (CSS) and overall (OS) survival observed among obese (BMI ≥ 30 kg/m^2^) patients with RCC. Prior studies have reported lower stage/grade tumors among obese patients to explain this. This study aimed to evaluate subcutaneous (SFA) and visceral fat area (VFA) associations with CSS, OS, tumor stage and grade among patients with non-metastatic clear cell RCC. **Methods:** Following IRB approval, patients undergoing nephrectomy for clear cell RCC between 2000 and 2023 were screened for inclusion. Eligible patients were those with non-metastatic disease and available preoperative imaging within 90 days of surgery. SFA and VFA were determined using mid-L3 imaging and standardized Hounsfield Unit thresholds. Multivariable Cox models evaluated factors associated with 5-year CSS and OS, and multivariable logistic regression models evaluated associations with pathologic stage (pT) 3–4 and Fuhrman grade 3–4 disease. **Results:** 400 patients were included. Higher SFA quartiles were independently associated with improved CSS (Q3 HR 0.21, *p* = 0.029; Q4 HR 0.24, *p* = 0.042) and OS (Q2-Q4 HR range 0.35–0.52, all *p* < 0.05) compared to Q1. VFA was not independently associated with OS and showed only an isolated association with CSS in the third quartile (HR 2.95, *p* = 0.041). Neither SFA nor VFA were independently associated with RCC stage or grade. **Conclusions:** Greater subcutaneous adiposity was associated with improved 5-year CSS/OS, whereas visceral adiposity did not demonstrate consistent associations with survival outcomes. These findings refine the obesity paradox and reflect the importance of fat distribution as a more specific risk factor than weight-based measures such as BMI alone.

## Simple Summary

Obesity is often associated with worse outcomes in cancer; however, the “obesity paradox” describes the observation that obesity may be associated with improved survival in patients with renal cell carcinoma (RCC). BMI is a crude measure of obesity as it does not distinguish different fat distributions. This study examined the associations between subcutaneous and visceral fat and survival outcomes in non-metastatic clear cell RCC. Greater subcutaneous fat was associated with improved cancer-specific and overall survival, whereas visceral fat demonstrated inconsistent associations with survival outcomes. These findings suggest that the location of fat in the body may be more important than overall body weight in understanding the obesity paradox in RCC.

## 1. Introduction

Obesity, defined as a body mass index (BMI) ≥30 kg/m^2^, is implicated in the development of various malignancies, including renal cell carcinoma (RCC) [1,2,3]. Epidemiologic studies have consistently demonstrated an association between obesity and increased RCC incidence [1,2,3]. Paradoxically, obesity has been associated with apparent cancer-specific (CSS) and overall (OS) survival benefits among patients with RCC [4,5]. This finding, termed the “obesity paradox,” remains controversial due to the conventional metabolic/systemic detriments associated with increased adiposity, particularly with respect to the prevalence of visceral fat [6,7]. Posited explanations for this observation have included the observation that lower stage/grade tumors are more prevalent among patients with greater adiposity and/or BMI values [8,9].

Importantly, several studies thus far have argued against the aforementioned paradox, attributing survival associations to variations in lean body mass as opposed to adiposity [10,11]. Given that BMI does not discriminate body composition, further research aimed at quantifying how variation in precisely quantified body composition is associated with survival is important in risk stratification/prognostication [11,12]. One such method of estimating body composition includes the segmentation of routine cross-sectional imaging in patients with RCC, which allows for the analysis of specific fat compartments [13]. This study aims to evaluate whether subcutaneous and visceral fat are independently associated with CSS, OS, tumor stage, and Fuhrman grade among patients with non-metastatic clear cell RCC (ccRCC).

## 2. Methods

Following institutional review board (IRB) approval, the nephrectomy database at Emory University was retrospectively reviewed for patients who underwent nephrectomy between 2000 and 2023. Eligible patients were defined as those aged ≥18 years with non-metastatic clear cell RCC (T1-4N0M0) who underwent nephrectomy with available CT imaging within 90 days preoperatively. Patients with known pathologic nodal spread or metastatic disease at the time of surgery were excluded. Patient clinical and demographic variables collected were age at nephrectomy, sex, race, Eastern Cooperative Oncology Group (ECOG) status, body mass index (BMI; kg/m^2^), Charlson Comorbidity Index (CCI) score, smoking status, type of nephrectomy, pathologic tumor stage (pT), Fuhrman grade, subcutaneous fat area (SFA), visceral fat area (VFA), death due to RCC within five years, and death due to any cause within five years.

**Figure 1:**
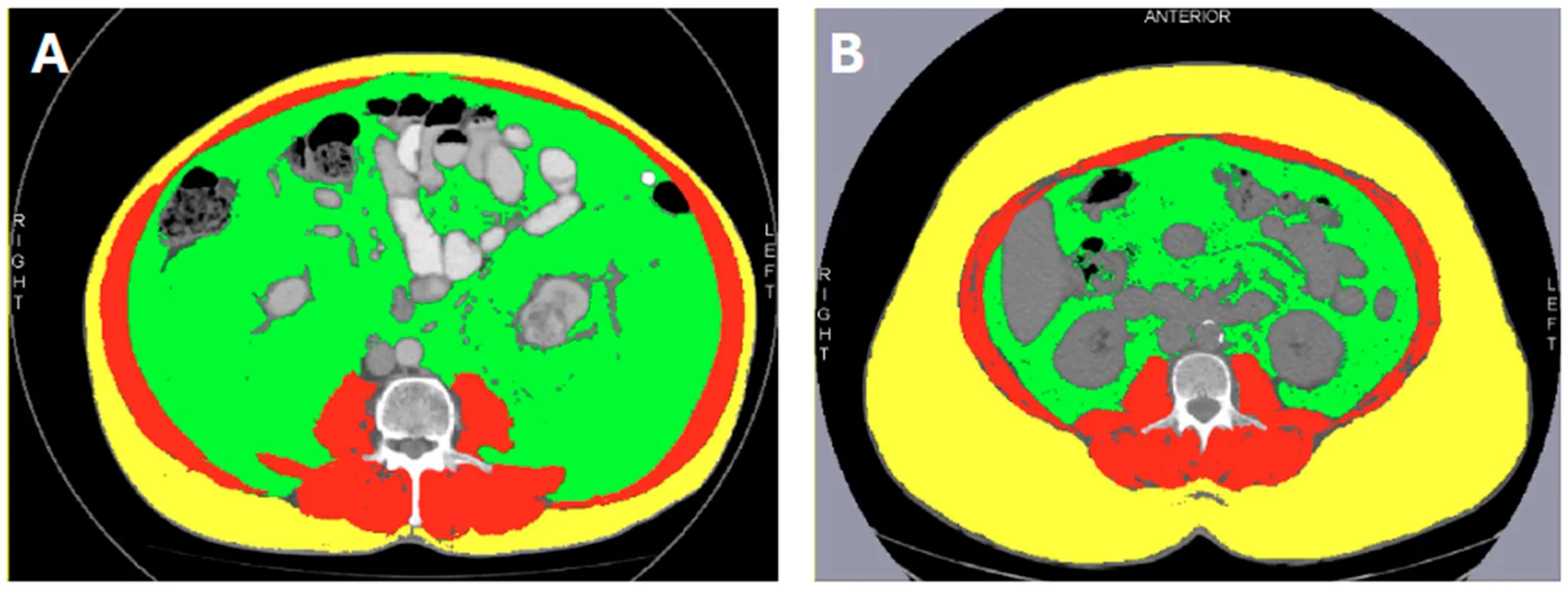
Examples of axial L3 image segmentation per Steele et al. guidance. Visceral fat area (VFA) is shown in green, subcutaneous fat area (SFA) shown in yellow, and skeletal muscle shown in red. (**A**) Patient with predominantly visceral adiposity. (**B**) Patient with predominantly subcutaneous adiposity.

SFA and VFA were determined using axial CT images taken within 90 days of surgery to ensure concordance with body composition at the time of nephrectomy [10,13,14]. Mid-L3 vertebral level axial slices were then identified and segmented using various Hounsfield unit (HU) thresholds, Slice-O-Matic 5.0 software (Tomovision, QC, Canada), and Horos (Nimble Co LLC d/b/a Purview, Annapolis, MS, USA) as described by Steele et al. [13]. The L3 level was selected given its established reliability as a single-slice surrogate for total body adiposity and skeletal muscle mass in body composition research [13]. HU thresholds of −150 to −50 were used to delineate visceral fat from other abdominal/luminal contents and calculate a visceral fat area (VFA), and thresholds of −190 to −30 units subsequently characterized subcutaneous fat area (SFA) in a similar manner. Figure 1 depicts two segmentation examples, one patient with significant visceral and limited subcutaneous adiposity (Figure 1A) and another with significant subcutaneous and limited visceral adiposity (Figure 1B).

Primary study endpoints were 5-year cancer-specific (CSS) and overall survival (OS) in line with cohort median follow-up. Secondary study endpoints included analyzing factors independently associated with elevated tumor stage (pT 3–4) and grade (Fuhrman 3–4). Categorical variables were reported as total counts with percentages, while continuous variables were reported as median values with interquartile ranges. Multivariable Cox proportional hazards models were used to evaluate factors independently associated with 5-year OS and CSS, adjusting for prognostic covariates in line with prior studies evaluating survival among RCC patients [8,10]. Tumor stage and grade were analyzed as continuous variables to prevent model overfitting and accurately capture their prognostic significance. Factors independently associated with elevated tumor stage (T3/4) and tumor grade (Fuhrman 3/4) were then analyzed using multivariable logistic regression models adjusting for the aforementioned covariates, selected based on prior literature, except stage and/or grade [8,15]. Complete-case analysis was performed for all multivariable models. All statistical tests were two-sided, and a significance threshold of *p* < 0.05 was used to determine statistical significance. Statistical analyses were performed using SPSS Statistics v31 (IBM Corp., Armonk, NY, USA).

## 3. Results

In total, 400 patients were included; 176 (44.0%) were ≥65 years old and 141 (35.3%) were female. Of our cohort, 110 (27.5%) patients were Black, and 182 (45.5%) met BMI criteria for obesity (BMI ≥ 30 kg/m^2^). The majority of patients (184, 46.0%) had Fuhrman grade 3 disease and pT1 disease (188, 47.0%). Cohort median (IQR) SFA and VFA were 226.3 (153.2–344.5) cm^2^ and 197.0 (114.6–299.1) cm^2^, respectively. At a median (IQR) cohort follow-up of 5.0 (2.2–7.5) years, 47 (11.8%) patients had a cancer-related death and 122 (30.5%) had a death due to any cause. Total cohort descriptive statistics are detailed in Table 1. SFA and VFA median (IQR) quartiles and corresponding BMI ranges are illustrated in Supplemental Table S1.

### 3.1. SFA, VFA, and 5-Year CSS

**Figure 2:**
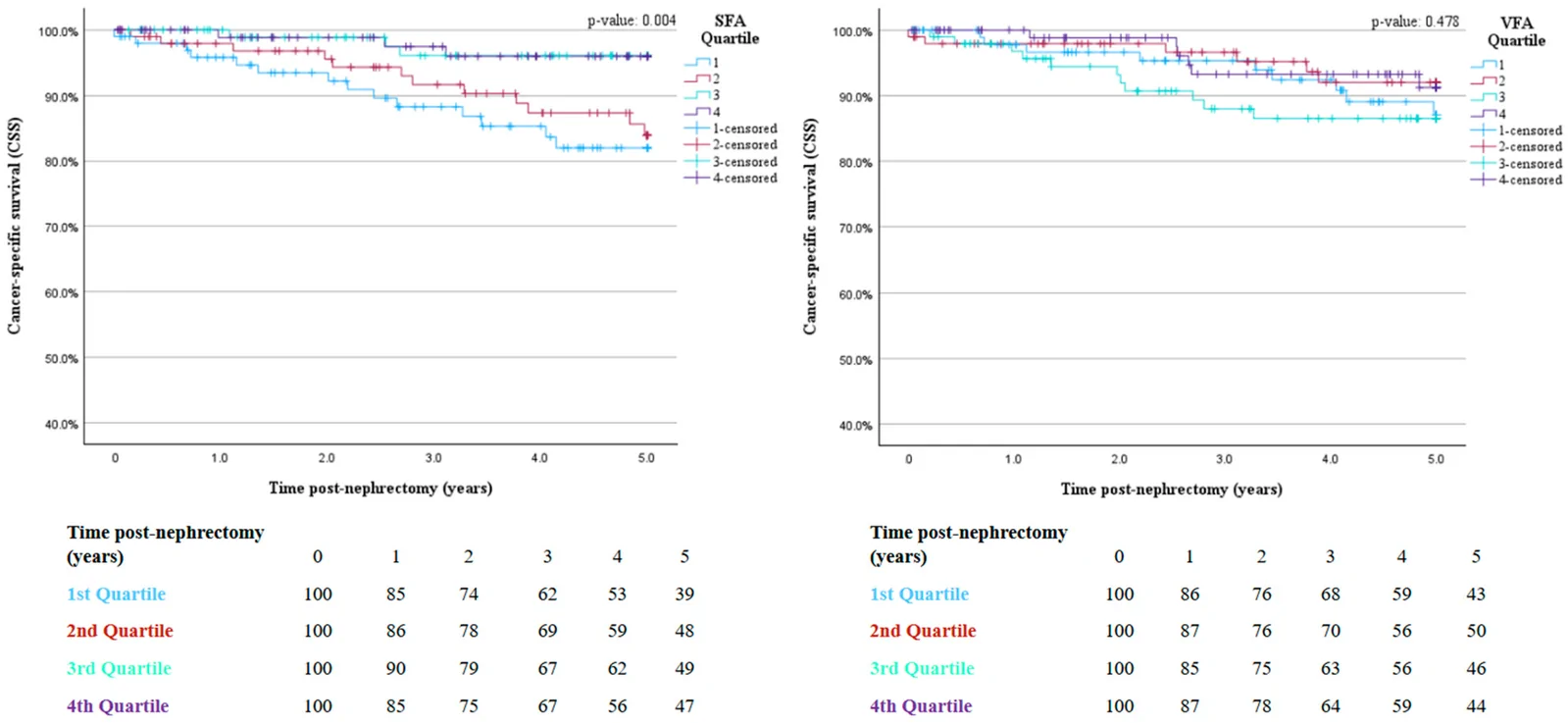
SFA and VFA 5-year CSS Kaplan–Meier curves and number at-risk tables.

Patients in the third and fourth quartiles of SFA demonstrated a 79% (HR 0.21, 95%CI 0.05–0.85, *p* = 0.029) and 76% (HR 0.24, 95%CI 0.06–0.94, *p* = 0.042) lower hazard of cancer-specific mortality risk compared to the first quartile, respectively. Conversely, the third quartile of VFA was associated with a 2.95-fold increase in 5-year cancer-specific mortality (HR 2.95, 95% CI 1.04–8.31, *p* = 0.041). Fuhrman grade was also independently associated with a 2.42-fold (HR 2.42, 95% CI 1.27–4.63, *p* = 0.007) per-unit increase in cancer-specific mortality risk. All 5-year CSS multivariable Cox proportional hazards model results are depicted in Table 2. Kaplan–Meier curves demonstrating cancer-specific survival across SFA and VFA quartiles are illustrated in Figure 2.

### 3.2. SFA, VFA, and 5-Year OS

**Figure 3:**
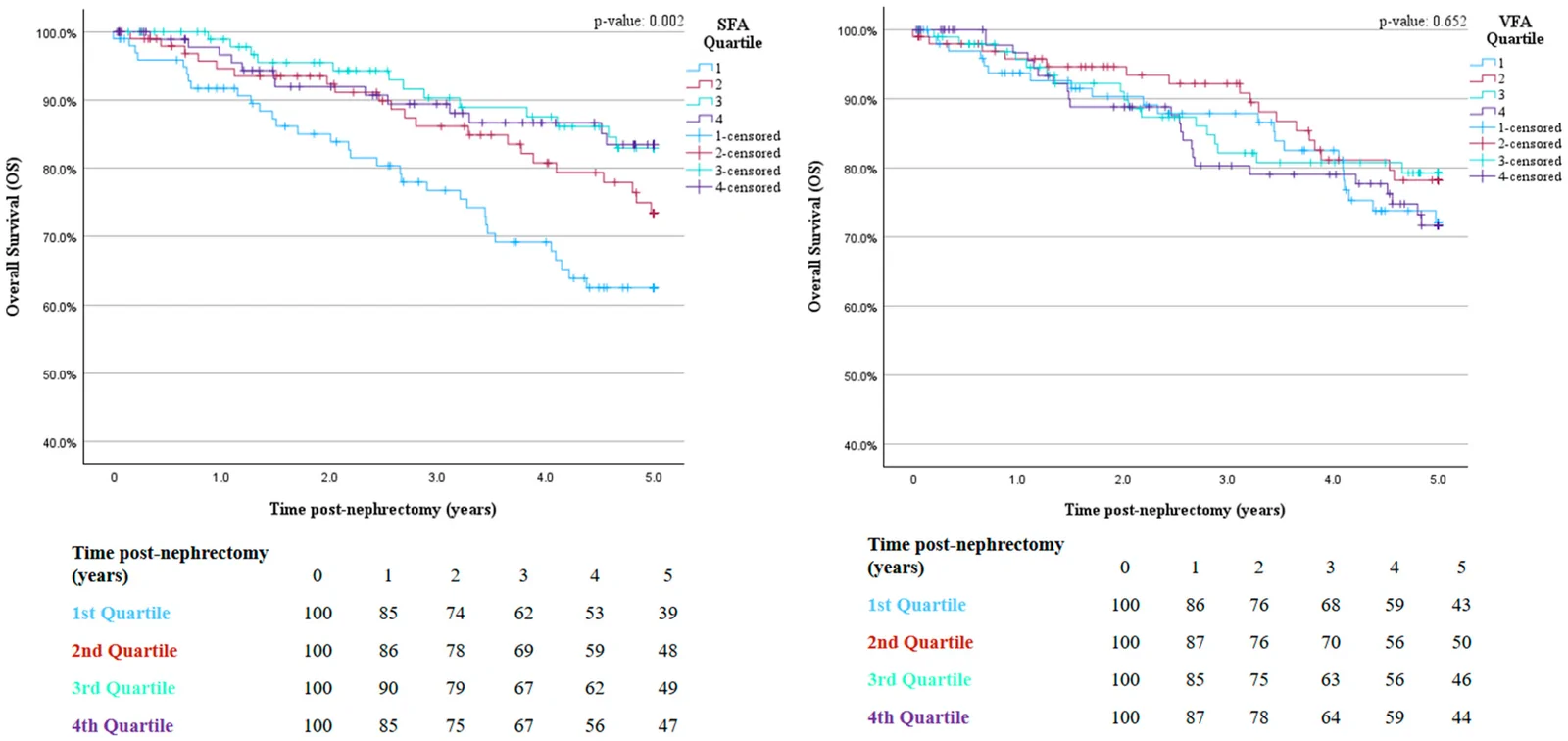
SFA and VFA 5-year OS Kaplan–Meier curves and number-at-risk tables at each timepoint beneath.

Patients in the second and third quartiles of SFA demonstrated a 0.48-fold (HR 0.52, 95% CI 0.27–0.99, *p* = 0.047) and 0.65-fold (HR 0.35, 95% CI 0.17–0.73, *p* = 0.005) decrease in 5-year all-cause mortality risk compared to the first quartile, respectively. Furthermore, patients with SFA values in the fourth quartile demonstrated a 0.62-fold (HR 0.38, 95% CI 0.18–0.81, *p* = 0.013) decrease in 5-year all-cause mortality risk compared to those in the first quartile. In contrast, VFA quartiles were not associated with overall survival (*p* > 0.05). Older age (≥65 years) was associated with a 1.99-fold (HR 1.99, 95% CI 1.21–3.27, *p* = 0.006) increased risk of 5-year overall mortality. Similarly, an ECOG ≥ 1 and CCI of ≥2 were significantly associated with a 2.30-fold (HR 2.30, 95% CI 1.29–4.08, *p* = 0.004) and 2.78-fold (HR 2.78, 95% CI 1.22–6.29, *p* = 0.014) increase in 5-year mortality risk. Finally, each increase in pT stage was also independently associated with a 1.78-fold (HR 1.78, 95% CI 1.29–2.46, *p* = <0.001) increase in mortality risk within 5 years of surgery. Multivariable Cox proportional hazards model results are depicted in Table 2. Kaplan–Meier curves demonstrating overall survival across SFA and VFA quartiles are illustrated in Figure 3.

### 3.3. SFA, VFA, and Tumor Stage and Grade

Neither SFA nor VFA were significantly associated with elevated grade (Fuhrman 3–4) or pT stage (pT 3–4). In contrast, older age (≥65) was associated with a 1.63-fold (OR 1.63, 95% CI 1.03–2.58, *p* = 0.036) increased odds of grade 3–4 disease and a 2.05-fold (OR 2.05, 95% CI 1.33–3.17, *p* < 0.001) increased odds of pT stage 3–4 disease. Multivariable logistic regression results analyzing factors independently associated with elevated tumor stage and grade are depicted in Table 3.

## 4. Discussion

Through the characterization of fat-specific survival associations, this study sheds light on the much-debated “obesity paradox” and notes several novel risk associations in the context of localized clear cell renal cell carcinoma. Among a racially diverse large cohort of patients with non-metastatic ccRCC, increasing subcutaneous fat was independently associated with reductions in both cancer-specific and overall mortality, whereas visceral fat demonstrated an isolated association with increased cancer-specific mortality. Importantly, neither visceral nor subcutaneous fat quartiles were associated with elevated tumor stage and grade in this study, alluding to alternative survival mechanisms.

Patients with subcutaneous fat area measurements in the third (CSS HR 0.21, 95% CI 0.05–0.85, *p* = 0.029) and fourth quartiles (CSS HR 0.24, 95% CI 0.06–0.94, *p* = 0.042) demonstrated a marked 5-year CSS benefit in this study. While this finding remains relatively novel among patients with non-metastatic ccRCC, similar findings have been documented in the broader oncologic setting [16,17]. Proposed hypotheses for improved survival among patients with elevated BMI have included indolent tumor stage/grade associations not observed with either subcutaneous or visceral fat in this study [17]. It is possible that patients with non-metastatic ccRCC and greater energy reserves indicated by elevated SFA exhibit non-stage/grade-related tumor biology differences such as reductions in systemic inflammatory burden, a purely hypothetical claim. Visceral fat area measurements in the third quartile were associated with a near three-fold (CSS HR 2.95, 95% CI 1.04–8.31, *p* = 0.041) increase in 5-year cancer-specific mortality; however, this isolated finding should be interpreted cautiously given the absence of significant associations in neighboring quartiles. Prior studies have associated visceral fat with elevated tumor stage/grade not observed in this analysis [18,19,20]. Irrespective, these findings require validation and suggest that all fat may not be created equal. Prior work has established visceral and subcutaneous fat as distinct metabolic and inflammatory phenotypes [21,22]. Our findings extend this framework to non-metastatic ccRCC, suggesting that fat distribution, rather than total mass, carries prognostic implications in non-metastatic ccRCC.

Similarly, patients with SFA measurements in the second (OS HR 0.52, 95% CI 0.27–0.99, *p* = 0.047), third (HR 0.35, 95% CI 0.17–0.73, *p* = 0.005) and fourth (HR 0.38, 95% CI 0.18–0.81, *p* = 0.013) quartiles all demonstrated marked improvements in 5-year OS after adjusting for prognostic confounders. This finding reinforces cancer-related survival benefits and remains novel in the context of non-metastatic ccRCC. Several hypotheses may explain the observed survival associations: first, given that subcutaneous fat is a proxy measure of cancer-related cachexia, it may be the case that patients with greater subcutaneous adiposity have less aggressive non-stage/grade-related cancer biology and associated systemic inflammation [23,24,25]. Such characteristics may be subvisual, such as pathomic/radiomic features that have been associated with cancer-related outcomes among patients with RCC [26,27]. Another potential explanation may include the fact that patients with greater indolent nutritional reserve have greater functional capacity and are therefore able to withstand the burden of RCC and insult of surgery. This is supported by prior work demonstrating associations between body composition and perioperative outcomes among patients with RCC, suggesting that nutritional reserve may influence recovery independent of oncologic risk [14,28]. Prior studies have shown varying associations between visceral fat and survival in localized RCC, with some demonstrating that greater visceral fat is associated with better overall and recurrence-free survival [29,30], and others showing no association between visceral fat and mortality [31]. Interestingly, no associations between visceral fat and OS were observed in this study, potentially due to the influence of subcutaneous adiposity/their collinearity. The validation of these hypotheses and our findings are warranted prior to their acceptance.

This study has several limitations. Firstly, as a single-center retrospective study, generalizability is limited, causality cannot be determined, and there is potential for selection bias. Second, the use of single-slice visceral and subcutaneous fat areas may not fully capture total-body adiposity or regional fat distribution, including appendicular and peripheral fat stores. Though single-slice measurements are commonly used and validated in body composition research, they may not reflect the complexity of whole-body fat composition. Third, this study did not analyze the interaction between subcutaneous and visceral fat or report on non-stage/grade outcomes, limiting conclusions regarding the broader biological mechanisms underlying these associations. Fourth, due to the novel nature of certain findings within localized RCC, several unvalidated hypotheses warrant further investigation. Finally, there exists the potential for residual confounding, including temporal confounding related to changes in clinical practice, diagnostic approaches, and treatment patterns over the study period. Additionally, several potentially relevant variables were not available for analysis, such as skeletal muscle volume/sarcopenia, non-muscular lean body mass, intramuscular fat, and height values, which may influence body composition assessment and survival outcomes [10,28,32].

## 5. Conclusions

In this study of patients undergoing nephrectomy for non-metastatic clear cell RCC, visceral and subcutaneous fat areas measured on axial CT imaging were differentially associated with cancer-specific and overall survival. Higher SFA quartiles were associated with improved cancer-specific and overall survival, while VFA quartiles demonstrated no association with overall survival and only an isolated association with cancer-specific survival. These findings further inform considerations regarding the underpinnings of the observed “obesity paradox” in RCC, highlighting the importance of fat distribution rather than total adiposity when prognosticating survival. Future studies are warranted to validate these findings and to further investigate the roles of subcutaneous and visceral fat across other malignancies.

## Figures and Tables

**Table 1:** Cohort clinical and demographic characteristics.

Covariate	Subgroup	Total (N = 400)
Age (years)	<65	224 (56.0%)
	≥65	176 (44.0%)
	Median (IQR)	62.75 (53.99–69.94)
Sex	Female	141 (35.3%)
	Male	259 (64.8%)
Race	White	265 (66.3%)
	Black	110 (27.5%)
	Other/Unknown	25 (6.3%)
ECOG	0	349 (87.3%)
	≥1	49 (12.3)
	Unknown	2 (0.5%)
BMI (kg/m^2^)	Non-obese (<30)	217 (54.3%)
	Obese (≥30)	182 (45.5%)
	Unknown	1 (0.3%)
	Median (IQR)	29.30 (26.00–34.20)
CCI	0	100 (25.0%)
	1	52 (13.0%)
	≥2	239 (59.8%)
	Unknown	9 (2.3%)
Smoking status	Never	222 (55.5%)
	Current/Former	174 (43.5%)
	Unknown	4 (1.0%)
Nephrectomy type	Radical	270 (67.5%)
	Partial	129 (32.3%)
	Unknown	1 (0.3%)
pT	1	188 (47.0%)
	2	19 (4.8%)
	3	180 (45.0%)
	4	6 (1.5%)
	Unknown	7 (1.8%)
Fuhrman grade	1	5 (1.3%)
	2	132 (33.0%)
	3	184 (46.0%)
	4	69 (17.3%)
	Unknown	10 (2.5%)
SFA (cm^2^)	Median (IQR)	226.3 (153.2–344.5)
VFA (cm^2^)	Median (IQR)	197.0 (114.6–299.1)
Follow-up (years)	Median (IQR)	5.0 (2.2–7.5)
Cancer-related death	No	353 (88.2%)
	Yes	47 (11.8%)
Death due to any cause	No	278 (69.5%)
	Yes	122 (30.5%)

Abbreviations: Eastern Cooperative Oncology Group number (ECOG); body mass index (BMI; kg/m^2^); Charlson Comorbidity Index number (CCI); pathologic T-stage (pT); interquartile range (IQR); subcutaneous fat area (SFA); visceral fat area (VFA).

**Table 2:** Multivariable Cox proportional hazards model results depicting factors independently associated with 5-year CSS and OS.

Covariate	Subgroup	5-y CSS HR (95% CI)	*p*-Value *	5-Year OS HR (95% CI)	*p*-Value *
Age	≥65	1.83 (0.82–4.09)	0.140	**1.99 (1.21–3.27)**	**0.006**
	<65	Reference		**Reference**	
Race	Other/Unknown	1.73 (0.47–6.38)	0.407	1.95 (0.83–4.59)	0.123
	Black	1.46 (0.55–3.86)	0.442	1.73 (0.98–3.03)	0.055
	White	Reference		Reference	
ECOG	≥1	2.13 (0.78–5.76)	0.586	**2.30 (1.29–4.08)**	**0.004**
	0	Reference		**Reference**	
CCI	≥2	1.66 (0.58–4.72)	0.337	**2.78 (1.22–6.29)**	**0.014**
	1	1.31 (0.27–6.23)	0.736	1.48 (0.47–4.60)	0.494
	0	Reference		**Reference**	
Smoking status	Current/Former	0.83 (0.37–1.86)	0.653	1.13 (0.70–1.84)	0.601
	Never	Reference		Reference	
Nephrectomy type	Partial	0.64 (0.13–3.20)	0.591	0.71 (0.32–1.51)	0.373
	Radical	Reference		Reference	
Fuhrman Grade **	Grades 1–4	**2.42 (1.27–4.63)**	**0.007**	1.10 (0.76–1.60)	0.582
pT **	1–4	**2.28 (1.19–4.36)**	**0.013**	**1.78 (1.29–2.46)**	**<0.001**
SFA Quartile	4	**0.24 (0.06–0.94)**	**0.042**	**0.38 (0.18–0.81)**	**0.013**
	3	**0.21 (0.05–0.85)**	**0.029**	**0.35 (0.17–0.73)**	**0.005**
	2	0.79 (0.31–2.01)	0.627	**0.52 (0.27–0.99)**	**0.047**
	1	**Reference**		**Reference**	
VFA Quartile	4	1.26 (0.39–4.11)	0.693	1.66 (0.84–3.31)	0.144
	3	**2.95 (1.04–8.31)**	**0.041**	1.30 (0.627–2.73)	0.474
	2	1.10 (0.29–4.17)	0.885	1.00 (0.47–2.16)	0.982
	1	Reference		Reference	

* Statistically significant (*p* < 0.05) results are in bold. ** Continuous variables. Abbreviations: cancer-specific survival (CSS); overall survival (OS); hazard ratio (HR); 95% Confidence Interval (95% CI); Eastern Cooperative Oncology Group Status (ECOG); Charlson Comorbidity Index Score (CCI); pathologic t-stage (pT); subcutaneous fat area (SFA); visceral fat area (VFA).

**Table 3:** Multivariable logistic regression results depicting factors independently associated with elevated tumor stage and grade.

Covariate	Subgroup	Grade 3–4 OR (95% CI)	*p*-Value *	pT 3–4 OR (95% CI)	*p*-Value *
Age	≥65	**1.63 (1.03–2.58)**	**0.036**	**2.05 (1.33–3.17)**	**<0.001**
	<65	**Reference**		**Reference**	
Race	Other/Unknown	2.01 (0.69–5.82)	0.198	2.02 (0.80–5.14)	0.140
	Black	0.91 (0.54–1.54)	0.734	0.70 (0.42–1.16)	0.168
	White	Reference		Reference	
ECOG	≥1	0.76 (0.39–1.47)	0.408	1.26 (0.66–2.43)	0.483
	0	Reference		Reference	
CCI	≥2	1.27 (0.74–2.16)	0.392	1.05 (0.62–1.78)	0.848
	1	1.09 (0.52–2.29)	0.817	0.66 (0.32–1.37)	0.265
	0	Reference		Reference	
Smoking status	Current/Former	1.46 (0.93–2.30)	0.100	1.08 (0.70–1.66)	0.733
	Never	Reference		Reference	
SFA Quartile	4	0.73 (0.36–1.47)	0.383	0.81 (0.41–1.58)	0.532
	3	0.83 (0.42–1.63)	0.588	0.99 (0.52–1.86)	0.966
	2	0.68 (0.35–1.32)	0.250	0.85 (0.45–1.60)	0.608
	1	Reference		Reference	
VFA Quartile	4	1.30 (0.23–2.69)	0.486	0.62 (0.31–1.24)	0.174
	3	0.87 (0.44–1.71)	0.686	0.62 (0.32–1.19)	0.149
	2	0.70 (0.36–1.35)	0.282	0.76 (0.40–1.44)	0.399
	1	Reference		Reference	

* Statistically significant (*p* < 0.05) results are in bold. Abbreviations: Fuhrman grade 3 and 4 (Grade 3–4); pathologic t-stage (pT); hazard ratio (HR); 95% confidence interval (95% CI); Eastern Cooperative Oncology Group status (ECOG); Charlson Comorbidity Index Score (CCI); subcutaneous fat area (SFA); visceral fat area (VFA).

## Data Availability

The datasets presented in this article are not readily available because they are maintained within an institutional database and subject to privacy and data-sharing restrictions. Requests to access the datasets should be directed to the corresponding author.

## Supplementary Materials

The following supporting information can be downloaded at: https://www.mdpi.com/article/10.3390/cancers18162626/s1. Table S1. SFA and VFA quartile median (IQR) ranges and corresponding BMI values.
