# Supplementary material for: Subcutaneous Fat Is Associated with Improved Survival in Patients with Non-Metastatic Clear Cell Renal Cell Carcinoma: Dissecting the Obesity Paradox
Source: Cancers (Basel). 2026 Aug 14;18(16):2626. doi: 10.3390/cancers18162626 (PMC13510489; doi:10.3390/cancers18162626)
Supplement: Supplementary file 1 [file cancers-18-02626-s001.zip › cancers-4466755-supplementary.pdf]

**Supplemental Table S1.** SFA and VFA quartile median (IQR) ranges and corresponding BMI values.

| Covariate | Subcategory | Median (IQR) | Median BMI (IQR) | Total (N = 400) |
|---|---|---|---|---|
| VFA | | | | |
| | 1st Quartile | 69.70 (40.52 - 88.83) | 24.40 (21.80 - 28.30) | 100 |
| | 2nd Quartile | 159.00 (135.70 - 178.10) | 28.55 (26.40 - 32.05) | 100 |
| | 3rd Quartile | 236.00 (215.10 - 260.10) | 30.55 (27.10 - 35.00) | 100 |
| | 4th Quartile | 359.10 (326.30 - 421.50) | 34.25 (29.50 - 39.25) | 100 |
| SFA | | | | |
| | 1st Quartile | 113.80 (93.10 - 133.90) | 24.25 (21.70 - 26.92) | 100 |
| | 2nd Quartile | 186.90 (171.70 - 205.50) | 27.25 (25.82 - 30.10) | 100 |
| | 3rd Quartile | 276.90 (254.00 - 311.10) | 30.85 (28.82 - 33.37) | 100 |
| | 4th Quartile | 444.20 (388.60 - 527.80) | 37.70 (33.70 - 42.60) | 100 |

Abbreviations: Body mass index (BMI; kg/m2); Interquartile range (IQR); Visceral fat area (VFA; cm2); Subcutaneous fat area (SFA; cm2)
